# Food‐derived extracellular vesicles in the human gastrointestinal tract: Opportunities for personalised nutrition and targeted therapeutics

**DOI:** 10.1002/jex2.154

**Published:** 2024-05-02

**Authors:** Natalie P. Turner

**Affiliations:** ^1^ Faculty of Health Queensland University of Technology Kelvin Grove Queensland Australia

**Keywords:** EV biodistribution, EV therapeutics, EV uptake, Extracellular vesicles, food, health, nutrition

## Abstract

Food‐derived extracellular vesicles (FDEVs) such as those found in mammalian milk and plants are of great interest for both their health benefits and ability to act as biological nanocarriers. While the extracellular vesicle (EV) field is expanding rapidly to perform characterisation studies on FDEVs from plants, yeasts and bacteria, species‐specific differences in EV uptake and function in the human gastrointestinal (GI) tract are poorly understood. Moreover, the effects of food processing on the EV surfaceome and intraluminal content also raises questions surrounding biological viability once consumed. Here, I present a case for increasing community‐wide focus on understanding the cellular uptake of FDEVs from different animal, plant, yeast, and bacterial species and how this may impact their function in the human, which will have implications for human health and therapeutic strategies alike.

## 1 FOOD‐DERIVED EXTRACELLULAR VESICLES—THE FORGOTTEN SOLDIERS OF HUMAN HEALTH

Human health and nutrition have long revolved around the paradigm, ‘You are what you eat’, emphasising the necessity of a rich and varied diet for optimal health and longevity (Carpenter, 2003). Increasing awareness of the omnipresence of extracellular vesicles (EVs; 50–1000 nm) in food products (food‐derived EVs; FDEVs) such as milk (Pieters et al., 2015), plant matter (Nemati et al., 2022), fruit and vegetable juices (Mu et al., 2014), fermented products such as yoghurt, beer (Stensballe & Bennike, 2014), and wine (Kulig et al., 2023; Morales et al., 2021), and their importance in human health is an area of research that has been vastly overlooked. The term ‘EV’ is an umbrella term for particles of various sizes (30–10,000 nm) (van Niel et al., 2022), densities, modes of biogenesis, and specific functions, but in general terms refers to any lipid bilayer‐bound particle released from a cell that lacks a functional nucleus and therefore cannot replicate (Théry et al., 2018; Welsh et al., 2024). EVs of all classes, including the popular exosome (30–150 nm), which originates from the endocytic pathway, and ectosome (100–1000 nm), arising from direct budding of the cell plasma membrane, are recognised for their ability to relay important messages via molecular interactions at the EV surface and the transfer of molecular cargo to recipient cells in the local and distant extracellular milieu, which in turn elicits a cellular response (Buzás et al., 2018; Mathieu et al., 2019). FDEVs such as those derived from milk and the juices of fruits or vegetables have been found to possess anti‐inflammatory properties both in vitro (Tong et al., 2023; Zhang et al., 2016) and in vivo, as demonstrated in mouse models of colitis (Teng et al., 2018; Tong et al., 2021). Here, they can interact with the gut microbiota by way of micro(mi)RNA transfer, improve epithelial barrier integrity of the intestinal mucosal cell layers, enhance cellular regeneration, and protect against further inflammation of the gastrointestinal (GI) tract (Teng et al., 2018; Tong et al., 2023; Zhang et al., 2016). Even more astounding are the positive effects human milk EVs have on the infant gut; here, they are believed to offer protection against necrotising enterocolitis (W. Chen et al., 2021; Guo et al., 2022) and possess immunomodulatory properties critical for optimal health and development in the newborn (Arnett & Viney, 2014; Karra et al., 2022; Turner et al., 2023). Some of these health benefits could be due, in part, to their association with, or encapsulation of the milk proteins butyrophilin, lactadherin, and lactoferrin, although this requires validation (Turner et al., 2023).

Mechanisms of EV uptake include clathrin‐dependent and ‐independent mechanisms, which have been well‐described previously (Mulcahy et al., 2014), albeit the sorting process of internalised cargo and its fate is somewhat unknown (Mathieu et al., 2019). A recent perspective article speculating on the ability of EVs to enter and exit the circulatory system to participate in inter‐ and intracommunication between tissues and organ systems highlighted the gaps in knowledge regarding EV biodistribution, and challenges associated with cell‐ or tissue‐targeting (Iannotta et al., 2023). However, this was written from the point‐of‐view of systemic administration of EVs via intraperitoneal, intramuscular or subcutaneous routes for therapeutic delivery. Oral administration of FDEVs for similar purposes, such as those derived from human milk, cow milk, or plants is just starting to gain traction in the therapeutic space (Nemati et al., 2022; Tong et al., 2023), but we as a community still lack a comprehensive understanding of the way ingested FDEVs are dealt with once in the human GI tract. Most importantly in this context, the exact mechanisms of orally‐delivered EV uptake in cross‐kingdom models have not been extensively explored.

Numerous studies have analysed the proteomic, lipidomic and transcriptomic cargo of milk EVs from various species (Benmoussa et al., 2019; Buratta et al., 2023; van Herwijnen et al., 2016) and performed functional in vivo (Samuel et al., 2021; Tong et al., 2021) and in vitro studies (Karra et al., 2022; Tong et al., 2023); the purpose of these studies was not related to nutrition but rather to downstream therapeutic applications. What has not been taken into account is whether there are differences in the uptake of EVs from different sources and species, and how this may influence their function. Food processing such as pasteurisation and lyophilisation may also impact the structural integrity and surface profile of FDEVs found in store‐bought food products. We recently reported on the isolation of cow milk‐derived small EVs (<200 nm) from lyophilised infant formula products and identified significant reductions in the particle concentrations, particle yields, proteins and microRNAs compared to EVs enriched from unprocessed cow milk (Turner et al., 2023). Others have reported morphological changes and alterations to the signatures of EV‐associated protein markers in EVs enriched from commercial cow milk under homogenised, pasteurised or ultra heat‐treated conditions (Kleinjan et al., 2021).

From a functional perspective, studies performed in mice have revealed that EVs from ginger, carrots, grapes and grapefruit are taken up by resident macrophages in the GI tract (Mu et al., 2014), whereas milk‐derived EVs cross into the GI epithelium (Tong et al., 2023). Understanding cross‐species differences in FDEV uptake, the sorting of internalised cargo, communication between the GI tract and other organ systems, and the expression of surface markers responsible for driving specific modalities of cellular uptake are critical for not only improving food products in the interest of human health, but will contribute to the development of orally‐delivered therapeutics (Cieślik et al., 2022; Donoso‐Meneses et al., 2023).

## 2 MILK PROCESSING – FRIEND OR FOE TO THE MILK EV?

There is a plethora of ways in which EVs from milk (or alternative food products, biofluids or conditioned cell media) can be enriched for downstream analyses or functional studies. These methods can be based on a number of different principles that exploit one or more of the biophysical properties of EVs. For example, differential centrifugation/ultracentrifugation (dUC), or density gradient ultracentrifugation (DGUC) leverage the size and/or density of EVs relative to cell debris, apoptotic bodies, microvesicles, soluble proteins or lipid particles to effectively deplete (although not completely) the non‐EV particles, and enrich for EVs that are usually <200 nm in diameter (Jeppesen et al., 2014; Théry et al., 2006). Size‐exclusion chromatography (SEC), on the other hand, separates particles purely based on their size and is particularly effective at removing soluble proteins that co‐isolate with EVs during dUC (Vaswani et al., 2019), but can also be used with a ‘neat’ sample that has been subjected to low‐speed centrifugation only, prior to SEC (Lobb et al., 2015). Ultrafiltration (UF) can concentrate and filter particles, the degree of which will depend on the specifics of the molecular weight cut‐off filter used (Vergauwen et al., 2017). The choice of EV isolation method should be dictated by the intended downstream applications, due in large part to the variability in purity, yield and concentration of particles resulting from each different method of EV enrichment (Hendrix et al., 2023). The Minimal Information for Studies of Extracellular Vesicles (MISEV) 2023 guidelines provides recommendations for working with milk EVs, which now includes multi‐step enrichment via combination methods (e.g., dUC + SEC), and pre‐processing for the removal of casein and milk fat globules – most commonly by acidification or the addition of EDTA – which will be crucial to maximising EV particle yield and purity (Welsh et al., 2024). It is worth mentioning here that the literature on large EV populations in milk is near non‐existent, perhaps due to technical challenges around separating casein micelle precipitate from large (>200 nm) ectosomes, which would be pelleted under similar centrifugal conditions (Benmoussa, Diallo, et al., 2019).

Following the isolation of EVs, appropriate characterisation experiments must be performed. These orthogonal experiments generally provide a measure of the success of the isolation method carried out by measuring EVs and co‐isolates. This can be achieved via detection of EV‐ and non‐EV‐associated protein markers via mass spectrometry(MS)‐based proteomics or western blot (WB), and measurements pertaining to particle size, concentration and morphology (Théry et al., 2018; Welsh et al., 2024). Methods typically employed to measure particle size and concentration are dynamic light scattering (DLS) or nanoparticle tracking analysis (NTA), flow cytometry (FCM), and morphological assessment by transmission electron microscopy (TEM), although these techniques are not without their limitations (Erdbrügger & Lannigan, 2016).

Human and cow milk EVs are the most widely studied FDEVs. In a nut shell, milk EVs are incredibly resilient, and although resistance to gastric digestion as demonstrated through in vivo models is a quality that has been observed in milk, plant EVs also display remarkable structural integrity under similar conditions (Kleinjan et al., 2021; Mu et al., 2014). Human milk EVs exhibit robustness in vitro at low pH with enzymatic digestion and heating to 37°C (Liao et al., 2017). However, when colorectal cancer cell (LIM1215)‐derived EVs were tested against bovine milk EVs in vitro under acidic conditions with boiling, only the milk EVs were stable; chelation of calcium from milk EV samples attenuated this stability, suggesting that milk EVs are innately equipped with resistance by way of surface binding to non‐vesicular molecules found in milk (Samuel et al., 2021). Indeed, interactions with particles long‐considered to be artefacts of relative unimportance or contamination in EV isolates, such as lipoproteins, are now under scrutiny for their association with the EV surface, which may facilitate EV function (Busatto et al., 2022; Lozano‐Andrés et al., 2023). Of note, a review highlighting the pathogenicity of pasteurised milk as responsible for increases in non‐communicable diseases of the 20th and 21st centuries, such as diabetes, obesity and various cancers, pointed at milk EVs (exosomes) as main contributors to the development of these conditions (Melnik & Schmitz, 2019). It must be appreciated that commercial milk processing has been shown to change the morphology and protein profile of bovine milk EVs significantly compared to unpasteurised milk (Kleinjan et al., 2021), which could represent a significant depletion of beneficial properties imbued by milk EVs, rather than a rise in toxicity from EV persistence through processing. Additionally, the authors did not consider changes to the total milk nutritional profile compared to unpasteurised milk, which should be further investigated for links with negative health outcomes.

**FIGURE 1:**
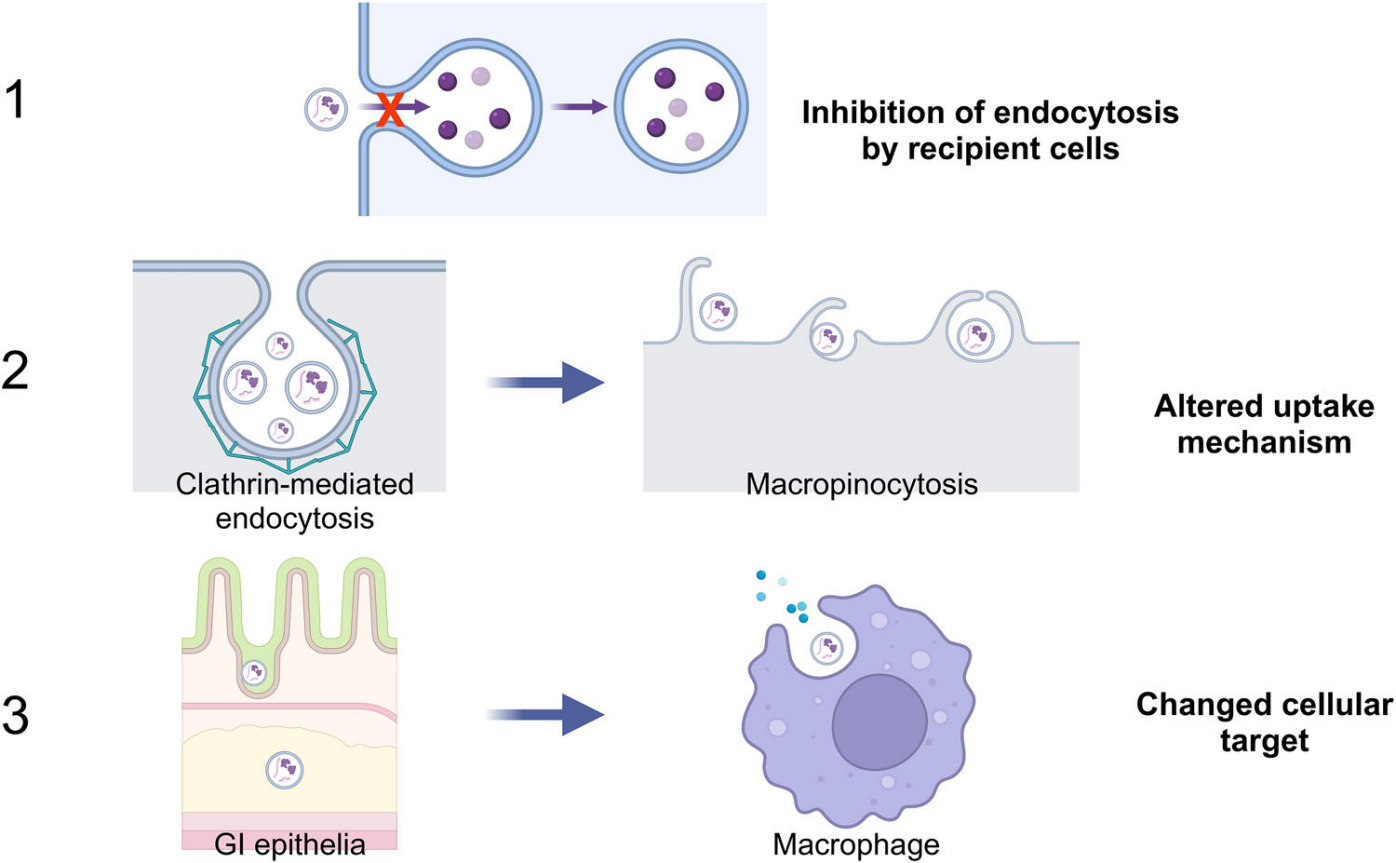
Pasteurisation and lyophilisation may change the surface profile of FDEVs. (1) Structural or conformational changes of the EV surface profile inhibits uptake by recipient cells. Reduced uptake of EVs limits their biological capabilities. (2) The mechanism of EV uptake by recipient cells is altered due to changes in EV membrane protein marker expression and thus the downstream fate of EV cargo is also altered. This has implications for the way EV cargo is processed and potentially changes or inhibits biodistribution. (3) The affinity for recipient cell type is affected such that EVs are taken up by, for example, immune cells rather than GI epithelial cells, also altering the fate of EV cargo. Created with BioRender.com. EV, extracellular vesicle; FDEVs, food‐derived extracellular vesicles.

**FIGURE 2:**
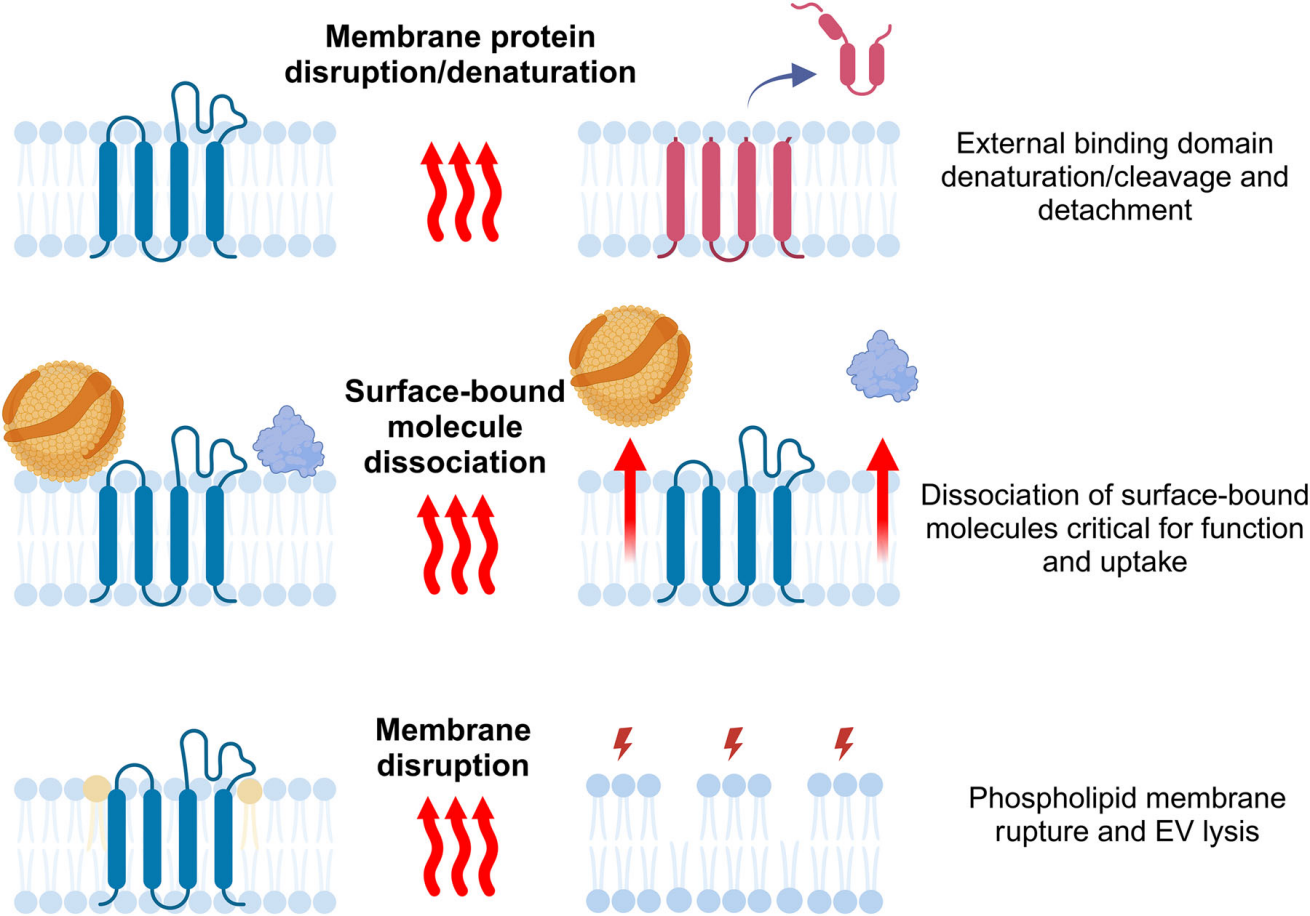
Schematic of possible changes occurring to the EV surface and structure due to pasteurisation and/or lyophilisation. Membrane protein disruption/denaturation: This may occur during food processing, such as the cleavage of external binding sites required for recognition and uptake by recipient cells. Surface‐bound molecule dissociation: Proteins or surface‐bound moieties that are externally associated only and not integrated into the EV membrane may dissociate, such as those acting as signalling factors for uptake, or others that contribute to their function, such as lipoproteins (Busatto et al., 2022; Lozano‐Andrés et al., 2023). Membrane disruption: Disruption of the EV membrane during heat treatment or lyophilisation may lead to lysis and loss of functional EV membrane molecules and intraluminal cargo, reducing the number of biologically active EVs in food products. Created with BioRender.com. EV, extracellular vesicle.

To summarise some of the gaps in knowledge surrounding food processing and its effects on milk EVs (and other FDEVs), it is not known whether pasteurisation or lyophilisation (1) ultimately affects the ability of milk EVs to be endocytosed in the GI tract, (2) alters the mode of internalisation or (3) changes the cellular target (Figures 1 and 2). Any or all of these alterations would likely have an impact on the ability of milk EVs to deliver their cargo to recipient cells, the biodistribution patterns of internalised cargo, and their overall therapeutic effect. Milk EV bioactives are still largely unexplored, so to gain a complete understanding of how food processing affects milk EVs and other FDEVs, comparison of pre‐processed (whole) milk and foods to the end‐products will be key to determining how and why FDEV structure and function is changed as a result. Studies of this nature are essential for determining factors contributing to reduced nutritional quality and would be hugely beneficial for understanding and improving processed milk products, including infant formulas (Leiferman et al., 2019; Turner et al., 2023). Some discrepancies also exist in the number of studies reporting the site of milk EV uptake; in vivo studies performed in mice and in vitro studies in human cell lines have reported milk EV uptake by intestinal epithelial cells (Liao et al., 2017; Samuel et al., 2021; Tong et al., 2023), however in vitro studies have also reported their uptake by macrophages (Izumi et al., 2015; Pieters et al., 2015). So, it seems the ability to interact with FDEVs in vitro and in vivo is shared by many cell types, including a variety of immune cells that reside in the intestinal lamina propria (Tong et al., 2023; Varol et al., 2010). Despite this, little is known regarding preferential uptake of EVs in the GI tract by different cell types, directed transport to various subcellular organelles and the GI immune response to EVs from a variety of species and sources. The EV isolation methodology utilised and degree of non‐EV cofactors present will undoubtedly contribute to observed variances between studies, which makes drawing definitive conclusions from the current available literature challenging and requires careful interpretation. Regardless, the factors driving differences in recipient cell‐type preference for EV uptake should be explored further, as this could direct targeted delivery of EVs in clinical applications.

## 3 WHAT'S DIFFERENT ABOUT PLANT EVS?

The extraction of EVs from plants requires washing to remove unwanted contaminants and particulates prior to blending, grinding or crushing (Lian et al., 2022; Zhang et al., 2016). Considering the latest updates to EV nomenclature, EVs isolated from plants starting with these methods will probably be closer to EV mimetics (Welsh et al., 2024), as the mechanical stress will result in cell disruption or extrusion of EVs rather than natural secretion from the cell. However, there are currently no specific recommendations for isolating or working with plant‐derived EVs (Welsh et al., 2024), and although dUC and DGUC seem to be the favoured EV isolation methods for plants to date (Ju et al., 2013; Zhang et al., 2016), there have been no comprehensive methodological reviews evaluating how different extraction methods affect the recovery or quality of plant EVs. Needless to say, studies of this kind will be required to advance this area of the EV field further. Similar to milk EVs, plant EVs extracted from the juice of fruits and vegetables have also been shown to pass through the upper GI tract intact (Mu et al., 2014). The size and composition of plant EVs in relation to mammalian EVs have been reviewed recently in detail (Lian et al., 2022), and suggests that they are not only biocompatible for therapeutic use in humans, but they possess many of the same qualities as mammalian EVs. On the other hand, the plant EV lipid membrane has a unique composition, which is believed to contribute to preferential uptake of plant EVs by resident gut bacteria (Teng et al., 2018) and macrophages (Wang et al., 2014). A comparison of some of the basic properties and characteristics of milk and plant EVs are shown in Table 1. What can be appreciated from this comparison is the difference in membrane composition, including proteins and lipids, which may heavily influence their interaction within the gut microenvironment and thus could direct their uptake via different routes or mechanisms. Milk Fat Globule‐Epidermal Growth Factor 8 (MFGE8), commonly known as lactadherin, is a secreted glycoprotein that most likely associates with milk EVs through selective binding to phosphatidylserine‐enriched membrane surfaces (Kamińska et al., 2018). MFGE8 binds to apoptotic cells to signal phagocytosis by macrophages, acting as an ‘eat me’ signal, therefore its binding to the EV surface could contribute to uptake of milk EVs in the human GI tract (Hanayama et al., 2002; Matsumoto et al., 2017). Whether the degree of MFGE8 binding with phosphatidylserine on the EV surface has an effect on this engulfment signal or directs alternative modes of internalisation is unknown. Further, these observations are currently limited to in vitro or systemic administration models, and in vivo models using orally delivered milk EVs provide evidence that milk EVs are first taken up by the GI epithelia rather than macrophages (Samuel et al., 2021; Tong et al., 2023). On the other hand, plant EVs, such as those derived from ginger, have membranes enriched in phosphatidic acid, which promotes their preferential uptake by gut microbiota (Teng et al., 2018). Studies on EVs extracted from ginger (Zhang et al., 2016) and grapes (Ju et al., 2013) demonstrate nuances in cell targeting and uptake, suggesting that there are inter‐species variations in the affinity for recipient cells among different plant varieties. When various plant‐derived EVs were cultured with the macrophage cell line RAW264.7, EVs extracted from ginger significantly increased the expression of anti‐inflammatory factors HO‐1, IL‐6 and IL‐10 compared to EVs extracted from carrot, grapes and grapefruit (Mu et al., 2014). However, macrophage and stem cell uptake of EVs was similar for all plant EVs under study, suggesting that the specific composition or cargoes of EVs from each plant type are responsible for the observed functional differences. While further studies are required to validate some of these speculative observations, it provides a starting point for elucidating the intricacies of FDEV composition and uptake from different animal and plant species. More thorough and extensive investigations will likely require the use of omics techniques and in particular, surface profiling strategies (Bauzá‐Martinez et al., 2022; Buzas, 2022; Buzás et al., 2018; Rai et al., 2021) to fully understand the interaction of plant EVs with the human gut microenvironment. Table 2 provides a summary of studies to highlight EV origin‐dependent differences in cellular uptake in vivo and in vitro.

**TABLE 1:** Basic properties and characteristics of milk and plant EVs.

EV origin	Size	Biogenesis	Membrane markers	Cargo
Milk EVs^a^	<200 nm	Endocytic pathway; direct budding from the plasma membrane	***Proteins***: CD9; CD63; CD81/82; FLOT‐1; MFGE8; ICAM1. ***Lipids***: Phosphatidylserine; sphingomyelin; ceramide; cholesterol; phosphatidylcholine; phosphatidylethanolamine	Endocytic components (Alix; TSG101; Syntenin‐1); Annexins; Rab proteins; GTPases; RNAs (mRNAs; miRNAs)
Plant EVs^b^	<500 nm	Exocyst‐positive organelles; endocytic pathway; others currently unknown	***Proteins***: Aquaporins. ***Lipids***: Phosphatidic acid; phosphatidylethanolamine; phosphatidylcholine	RNAs (*AG21*; *APS1*; *PRXIIC*; *HEL)*; small RNA binding proteins (AGO1; ANN1; ANN2; RH11; RH37); multivesicular body components (TET8/9); PEN1; HSP70

^a^ Benmoussa, Gotti et al. (2019); W. Chen et al. (2021); Turner et al. (2023); van Herwijnen et al. (2016).

^b^ He et al. (2021); Ju et al. (2013); Lian et al. (2022).

**TABLE 2:** Summary of studies related to EV uptake in in vivo (orally delivered) and in vitro models.

	Host—EV origin	Recipient	Model system	Uptake by cell‐type reported	EVs tracked to cell/organ systems	Method of EV isolation	EV characterisation
Mammalian EVs	Bovine – milk (Samuel et al., 2021)	Mouse	In vivo	GI epithelia	GI tract, kidney, liver, spleen, heart, lungs	dUC/DGUC	WB; MS; NTA; TEM
	Bovine and Human – milk (Tong et al., 2023)	Mouse	In vitro & in vivo	GI epithelia (Bovine)^a^	GI tract (colon)	dUC/DGUC/UF + CaCl_2_	WB; NTA; TEM
	Bovine – milk (Benmoussa, Diallo, et al., 2019)	Mouse	In vivo	GI epithelia (although not specifically stated)	NA	dUC + sodium citrate	‐
	Bovine – milk (Somiya et al., 2018)	Mouse	In vitro & in vivo	RAW264.7 cells (murine macrophage)	Macrophages	dUC + acetic acid	WB; MS; NTA; TEM
	Human – milk (Liao et al., 2017)	Human	In vitro	Human intestinal epithelial crypt‐like cells (HIEC)	GI epithelia and nucleus	dUC/UF/ ExoQuick	WB; immunoblot array; TEM
Plant EVs	Ginger – juice (Zhang et al., 2016)	Mouse and Human	In vitro & in vivo	RAW264.7 cells; Caco‐2BBE cells (human enterocytes)^b^; Colon‐26 cells (murine colon adenocarcinoma)	In vivo (mouse)—GI tract (colon) In vitro (mouse)—colonic epithelial cells; macrophages; dendritic cells (DCs)	dUC/DGUC	MS; DLS; TEM; AFM
	Grape – juice (Ju et al., 2013)	Mouse	In vitro & in vivo	CT26 cells (murine intestinal epithelia); GI epithelia	In vivo—GI tract (colon); LGR5 (colonic intestinal) stem cells. In vitro—GI epithelia and cytosol	dUC/DGUC	MS; DLS; cryo‐TEM; zeta potential
	Grape, ginger, grapefruit, carrot – juice (Mu et al., 2014)	Mouse	In vitro & in vivo	RAW264.7 cells; GI epithelia; intestinal macrophages	In vivo—GI tract (colon); lamina propria of small and large intestine; LGR5 stem cells In vitro—macrophages	dUC/DGUC	DLS; TEM; zeta potential
	Ginger, grapefruit—juice; engineered nanovectors derived from ginger and grapefruit EVs (Teng et al., 2018)	Mouse, Human, Bacteria (*Lactobacillus rhamnosus;* LGG).	In vitro & in vivo	Caco‐2 cells (human epithelial colorectal adenocarcinoma); MC‐38 cells (murine colon adenocarcinoma); LGG	In vivo—Duodenum; colon; liver In vitro—Mouse and Human epithelial cells; LGG	dUC/DGUC	MS; DLS; TEM; zeta potential
Fungal EVs	Baker's Yeast (*Saccharomyces Cerevisiae*) (Higuchi et al., 2023)	Mouse	In vitro	RAW264.7 cells; DC2.4 cells (mouse dendritic cells)	Macrophages; DCs	dUC	WB; DLS; zeta potential; AFM
	*Saccharomyces boulardii* CNCM I‐745 (Kulig et al., 2023)	Human	In vitro	THP‐1 cells (human monocytes)	NA	dUC	MS; NTA; TEM
	*Saccharomyces boulardii* CNCM I‐745 (Mierzejewska et al., 2023)	Human	In vitro	HT‐29 & HCT116 cells (human colorectal cancer cell lines); CCD841 (normal colon cells)	Cell membrane; cytoplasm	Sequential centrifugation/filtration/UF	MS; NTA; scanning TEM
Bacterial EVs	*Streptococcus salivarius* K12 (Kulig et al., 2023)	Human	In vitro	THP‐1 cells (human monocytes)	NA	dUC	MS; NTA; TEM
	*H. pylori 251; P. aeruginosa PA103 ΔpilA; S. Typhimurium SL1344; UPEC CFT073; P. gingivalis W50* (Bitto et al., 2017)	Human	In vitro	AGS (gastric adenocarcinoma cells)	EVs entered AGS cells; EV‐DNA co‐localised to cell nucleus (*P. aeruginosa* only)	dUC/DGUC	TEM; SRM
	*P. cedrina; P. panacis* (Choi et al., 2015)	Mouse	In vivo	Intestinal lamina propria	Liver; adipose tissue; skeletal muscle	UF; TFF; dUC	DLS; TEM

Abbreviations: AFM, atomic force microscopy; DLS, dynamic light scattering; DGUC, density gradient ultracentrifugation; dUC, differential centrifugation/ultracentrifugation; MS, mass spectrometry; NTA, nanoparticle tracking analysis; SRM, super resolution microscopy; TEM, transmission electron microscopy; TFF, tangential flow filtration; WB, Western blot.

^a^ Bovine and human milk EVs were both evaluated in vitro, while only bovine milk EVs were evaluated in vivo for practical reasons, as stated by the authors.

^b^ Caco‐2BBE cells were used to assess epithelial barrier function only.

## 4 FERMENTED FOODS AND THE MICROORGANISMS BEHIND THEM

Fermentation requiring the presence of yeast or bacterial species naturally gives rise to EVs from these microorganisms in the resulting food products. Beer, wine, and fermented dairy products are examples of this (Mierzejewska et al., 2023; Pérez Martínez et al., 2023; Voidarou et al., 2020); yet the number of studies on EVs from these sources is relatively scarce. Yeast and bacterial EV studies using mass spectrometry (MS) have identified heat shock protein family members and vacuolar proteins, suggesting that these proteins are widely conserved across species (Kulig et al., 2023; Mencher et al., 2020). In yeast strains, sorting of proteins into multi‐vesicular bodies (MVBs) occurs via the ESCRT pathway, however the exact mechanism is distinct from the mammalian ESCRT pathway (Bowers et al., 2004), and thus contributes to alterations in the cargo and composition of yeast EVs. The study and comparison of yeast to mammalian EV membranes is further confounded by the fact that homologues of the widely studied integral membrane proteins belonging to the tetraspanin family have not yet been identified in yeast (Jimenez‐Jimenez et al., 2019). Interaction between the resident gut microbiota, their associated vesicle‐types, and FDEVs is yet another important aspect, as milk EVs have been shown to improve gut cell morphology and influence the gut microbiome in in vivo models of ulcerative colitis (Du et al., 2022; Tong et al., 2021). Regarding mechanisms of uptake, EVs from *Saccharomyces cerevisiae* (Baker's yeast) were found to be taken up by murine immune cells by phagocytosis/macropinocytosis and clathrin‐mediated endocytosis (Higuchi et al., 2023), however whether this is the same in vivo and the exact yeast EV membrane components that contribute to uptake via these mechanisms are unknown. The interplay between gut microbiota, host, and FDEVs is complex and of significance in discerning uptake and eventual biodistribution of FDEV cargo in the human GI tract.

## 5 FOOD TECHNOLOGY – A TREASURE TROVE OF THE VESICULAR VARIETY

Venture capital‐backed start‐up companies such as BIOMILQ (https://www.biomilq.com/), Eden Brew (https://www.edenbrew.com.au/), and Eclipse (CSIRO; https://www.csiro.au/en/) (Mankad & Carter, 2022) use in vitro technologies to replicate food products or proteins. Specifically, precision fermentation utilised by the latter two companies involves the engineering of yeast species to create recombinant proteins, such as the bovine casein protein and human lactoferrin, with the goal of producing sustainable and nutritionally optimal food products for human consumption (Vanhercke & Colgrave, 2022). A rather fortuitous yet neglected byproduct of precision fermentation is the anticipated presence of yeast‐derived EVs in the litres of culture media required to sustain commercial engineered yeast strains. Utilisation of this culture media could prove invaluable for the further study of yeast EVs, especially considering alterations to the yeast genome and how this may affect their EV cargo. It also opens up opportunities to explore similar fermentation models in the context of mass production of EVs for therapeutic use – something that will only be possible by fully understanding their biogenesis, release, uptake and total membrane profile, including how they are endocytosed and interact with mammalian systems (Mierzejewska et al., 2023; Morales et al., 2021).

## 6 THERAPEUTICS: SLIGHTLY OFF‐TARGET?

Yeast and bacterial EVs have been studied for their potential as delivery vectors for vaccines or chemotherapeutic agents, with promising results (Bitto et al., 2017; Mierzejewska et al., 2023). Milk EVs are also attractive as therapeutic vehicles due to their high abundance and the ease in which they can be obtained, ability to be administered orally, ability to withstand low pH and high temperatures, and their natural anti‐inflammatory profile (Tong et al., 2023). There is still much to learn regarding the composition of human milk versus bovine milk EVs, particularly human milk EV‐specific surface protein markers, which may be critical to optimal cellular uptake in the human GI tract and direct the biodistribution of EVs and their cargo. Not limited to protein, microRNA, or lipids, the analysis of the bovine milk EV glycome and glycoproteome have shown promise for revealing specific glycosites to enhance targeted EV cargo delivery (W. Chen et al., 2020). Bovine milk EVs have low immunogenicity in humans, but as the field expands, milk EVs from other species such as goat (Mecocci et al., 2020) or yak (Gao et al., 2019) may be better suited for tolerance in humans, as both have been shown to possess similar desirable qualities to bovine milk EVs, although the number of milk EV studies in other mammalian/ruminant species is currently limited.

Engineered EVs, such as synthetic vesicles (SVs) or EV mimetics (Welsh et al., 2024), are becoming a popular choice of nanovector in the therapeutic space. Some of the key issues with engineered EVs, however, is the ability to identify and reproduce the most important aspects of biologically‐derived EVs that lead to their optimal uptake and function (Murphy et al., 2019), while guaranteeing scalability, safety, and reproducibility of EV production (Bonner et al., 2024; Ruan et al., 2022). Without extensive profiling of the EV surfaceome and appropriate incorporation of relevant surface moieties for directed cell targeting or uptake, one can only hazard a guess as to whether EV mimetics will indeed reach their target and be taken up by the recipient cells of interest to have the desired therapeutic effect, although the use of machine learning models has been proposed as a possible tool to aid in the EV design process (Rosso & Cauda, 2023). Factors contributing to EV clearance by macrophages (phosphatidylserine), or inhibition of clearance by macrophages (CD47), could be incorporated into EV mimetics for systemic administration to improve or direct uptake (Kamerkar et al., 2017; Matsumoto et al., 2017), but how these mechanisms are altered upon oral administration is yet to be established. Considering the potential for the development of personalised nutritional products, the overall therapeutic benefit is unlikely to be replicated in EV mimetics compared to viable biological sources without an exceptional understanding of the entirety of EV molecular and membrane cargo constituents and their relationship with the GI microenvironment.

Surprisingly, although the EV therapeutics field is exploding, it seems that the mode of therapeutic delivery has been driven towards subcutaneous, intraperitoneal or intravenous administration (Iannotta et al., 2023) of biological or synthetic nanocarriers. Knowing the challenges associated with targeting EVs to a specific cell or tissue type once they gain entry into systemic circulation, the concept of FDEVs as oral delivery vectors, which seem to be guaranteed to reach the GI tract intact to target a discrete population of cells, represents a significant opportunity that the EV community and commercial entities should consider very carefully.

## 7 CONCLUSION

The summary of information and ideas presented in this commentary have sought to force FDEVs into the spotlight as promising targets for boosting the nutritional value of food products and potential delivery vectors for therapeutics. To address the gaps in knowledge regarding mechanisms of uptake in cross‐kingdom models is likely quite simple in terms of experimental design, but ultimately it is the molecular interactions driving these processes that will enlighten the field as to what dictates downstream cargo processing for either degradation or propagation. Extensive and meticulous characterisation of EVs from mammalian, plant, fungal and bacterial origin, the impact of food processing, and the surface receptors or molecules that control FDEV uptake will be necessary steps to guide the field towards next‐generation therapeutics and nutritional products.

## AUTHOR CONTRIBUTIONS

**Natalie P. Turner**: Conceptualisation; data curation; visualization; writing—original draft; writing—review and editing.

## CONFLICT OF INTEREST STATEMENT

The author declares no competing financial interest.
